# Complete Genome Sequence of *Bacillus thuringiensis* Bacteriophage Smudge

**DOI:** 10.1128/genomeA.00572-16

**Published:** 2016-08-18

**Authors:** Jessica L. Cornell, Eileen Breslin, Zachary Schuhmacher, Madison Himelright, Cassandra Berluti, Charles Boyd, Rachel Carson, Elle Del Gallo, Caris Giessler, Benjamin Gilliam, Catherine Heatherly, Julius Nevin, Bryan Nguyen, Justin Nguyen, Jocelyn Parada, Blake Sutterfield, Muruj Tukruni, Louise Temple

**Affiliations:** Department of Integrated Science & Technology, James Madison University, Harrisonburg, Virginia, USA

## Abstract

Smudge, a bacteriophage enriched from soil using *Bacillus thuringiensis* DSM-350 as the host, had its complete genome sequenced. Smudge is a myovirus with a genome consisting of 292 genes and was identified as belonging to the C1 cluster of *Bacillus* phages.

## GENOME ANNOUNCEMENT

The bacteriophage Smudge was isolated on *Bacillus thuringiensis* DSM-350 as the host organism from soil samples taken from the James Madison University campus in Harrisonburg, VA, in 2015 (GPS coordinates 38°44′80.81″ N, 78°86′59.41″ W) by undergraduate researchers in collaboration with the SEA-PHAGES (Science Education Alliance-Phage Hunters Advancing Genomics and Evolutionary Science) program. Because the bacterium *B. thuringiensis* is safe for human use, even though it is commonly used as a biopesticide, it was safe for undergraduates to use in the research lab (1).

Smudge is a myovirus, as shown by the presence of contractile and extended tails. The extended tail was 49.7 ± 2.1 nm, and the contracted tail was 29.5 ± 1.5 nm. The symmetrical head diameter was 23.2 ± 2.4 nm. In this study, the sample soil was enriched using standard procedures to isolate a single phage. Phage genomic DNA was submitted to the Pittsburgh Bacteriophage Institute for Illumina Sequencing. About 50,000 reads of raw data were assembled into a single contig using Newbler (2) with 100-fold average depth of coverage. Gene prediction was completed using GeneMark (3) and Glimmer (4). Smudge was autoannotated using DNA Master (http://cobamide2.bio.pitt.edu/computer.htm) and all gene predictions were manually curated. Functions of proteins were predicted using protein BLAST (5), HHPRED, and Conserved Domains Database.

The Smudge genome consisted of 162,040 bp with a 38.8% GC content. Similar to other *Bacillus* phages, the genome contained a direct terminal repeat of 2,820 bp determined by the occurrence of double coverage with well-defined margins in the assembled contig. Smudge is most closely related to Megatron (KJ489401), with 98% query coverage (5). This finding and similarity to other published phages place Smudge in the C1 cluster (6). The number of predicted protein-coding regions found was 292, and no tRNA genes were present, as shown by Aragorn (7). Structural proteins included tail lysins, baseplate, tail sheath, tail fiber, major capsid, terminase, and prohead protease. Genes involved in DNA replication included DNA helicase, DNA primase, and DNA polymerase. Gene regulatory proteins included sigma factors, two helix-turn-helix binding proteins, and an I-Basl protein. Seven proteins involved in nucleic acid metabolism were adenylate kinase, thymidylate synthase, exonuclease, flavodoxin, thioredoxin, and two ribonucleotide diphosphate reductase beta subunits. The genome also was predicted to contain two endolysins and a holin. There were also two types of recombination proteins, Holliday junction resolvase and recombinase A. Aside from these genes, approximately 1,500 bp in the terminal repeats were noncoding; two hypothetical proteins were predicted in the remaining portion of the terminal repeats.

### Accession number(s).

The complete genome of bacteriophage Smudge is deposited in GenBank under the accession number KX156152.
